# Neural Correlates of Interval Timing Deficits in Schizophrenia

**DOI:** 10.3389/fnhum.2019.00009

**Published:** 2019-01-29

**Authors:** Ariel W. Snowden, Catalin V. Buhusi

**Affiliations:** Interdisciplinary Program in Neuroscience, Department of Psychology, Utah State University, Logan, UT, United States

**Keywords:** schizophrenia, interval timing, attention, cognitive dysfunction, working memory, neural correlates

## Abstract

Previous research has shown that schizophrenia (SZ) patients exhibit impairments in interval timing. The cause of timing impairments in SZ remains unknown but may be explained by a dysfunction in the fronto-striatal circuits. Although the current literature includes extensive behavioral data on timing impairments, there is limited focus on the neural correlates of timing in SZ. The neuroimaging literature included in the current review reports hypoactivation in the dorsal-lateral prefrontal cortex (DLPFC), supplementary motor area (SMA) and the basal ganglia (BG). Timing deficits and deficits in attention and working memory (WM) in SZ are likely due to a dysfunction of dopamine (DA) and gamma-aminobutyric acid (GABA) neurotransmission in the cortico-striatal-thalamo-cortical circuits, which are highly implicated in executive functioning and motor preparation.

## Introduction

*Schizophrenia* (SZ) is a complex, heterogeneous psychiatric disorder characterized by a broad range of symptoms including delusions, hallucinations, impaired cognitive functioning, disorganized speech and behavior (Patel et al., 2014). SZ patients show deficits in interval timing, perceiving durations in the seconds-to-minutes range (Densen, 1977; Lee et al., 2009). Table 1 shows the inconsistent pattern of *time perception* (TP) in SZ, possibly due to population heterogeneity and variability in illness duration (often not reported), symptom severity, and differential effects of typical vs. atypical antipsychotics. As temporal production tasks (Buhusi and Meck, 2010) often report less precision (greater variability) in time estimation (Carroll et al., 2009a,b; Roy et al., 2012; see also recent meta-analyses Ciullo et al., 2016; Thoenes and Oberfeld, 2017) and temporal estimation tasks often report overestimating durations (Densen, 1977; Wahl and Sieg, 1980; Tysk, 1983) differences in task demands may also account for inconsistent findings in SZ.

**Table 1:** Neuroimaging and behavioral paradigms used to assess time perception (TP) in schizophrenia (SZ) patients.

Article	Sample	Age (m ± SD)	Gender	Diagnosis	Medication	Onset	Task	Duration	Findings
Volz et al. (2001)	8 SZ 15 HC 1 SZA	25.3 ± 3.6 SZ(A) 31.7 ± 12.1 HC	M only	DSM-IV ICD-10	9 atypical No high-potency typical drugs 2 weeks prior	N/A	Duration discrimination	1 s, 1.4 s	↓ accuracy SZ ↓ DLPFC SZ
Ortuño et al. (2005)	11 SZ 10 HC	27.5 SZ 26.1 HC	10 M/1 F SZ 7 M/3 F HC	DSM-IV	8 unmedicated 3 taken off medication 1 month prior	N/A	Temporal reproduction	120 s	↓ accuracy SZ ns timing SZ vs. HC ↓ SMA SZ
Yoon et al. (2013)	18 SZ 19 HC	33.1 SZ 28.8 HC	66.7% M SZ 57.9% 19 HC	DSM-IV-TR	1 typical 17 atypical	N/A	Delayed-response Face WM paradigm	Stimulus presentation-1 s, 15 s delay, match discrimination	↓ accuracy SZ ↑ reaction time SZ ↓ PFC SZ ↓ BG SZ
Tysk (1983)	50 SZ 8 SPD 60 HC	34.8 SZ 34.1 SPD 37.0 HC	37 M/21 F SZ/SPD 26 M/34 F HC	DSM-III	neuroleptics drug type N/A	N/A	Temporal estimation Temporal production	Estimation: 1, 7.5, 17.5, 27.5 s Production: 10, 20, 30 s	↑ estimation SZ ↑ variability SZ ↓ production SZ ↑ clock speed
Densen (1977)	10 SZ 10 HC	19–46	N/A	Selected from chart diagnosis	N/A	N/A	Temporal estimation	5, 10, 30, 120 s	↑ estimation SZ ↑ clock speed
Carroll et al. (2009a)	32 SZ 31 HC	43.2 SZ 33.4 HC	22 M/10 F SZ 9 M/22 F HC	DSM-IV	7 typical 14 atypical	N/A	Temporal reproduction (Finger-tapping)	500 ms	↑ tapping SZ ↑ clock speed ↑ variability SZ
Roy et al. (2012)	25 SZ 25 HC	25.7 SZ 25.7 HC	24 M/1 F SZ 24 M/1 F HC	DSM-IV	23 atypical 2 unmedicated	5.9 yrs 1 yr min.	Temporal estimation Temporal reproduction	800, 1,600, 2,400 ms	ns clock speed SZ vs. HC ↑ variability reproduction SZ
Carroll et al. (2009b)	28 SZ 31 HC	40.8 SZ 37.8 HC	21 M/7 F SZ 11 M/20 F HC	DSM-IV	6 typical 12 atypical 1 unmedicated 8 N/A	N/A	Temporal bisection	0.3 s, 0.6 s anchors 3, 6 s anchors	↓ accuracy SZ ns PSE SZ vs. HC ↑ variability SZ subsecond ↑ variability SZ suprasecond
Wahl and Sieg (1980)	26 SZ 26 HC	39.8 ± 7.9 SZ 18.9 ± 1.9 HC	12 M/14 F SZ 19 M/7 F	N/A	N/A	N/A	Temporal estimation	5, 15, 30, 60 s	↓ accuracy SZ ↑ estimation SZ ↑ clock speed

*Duration discrimination: participants are presented with two durations and determine which duration is longer/shorter. Temporal production: a stimulus is presented and response is required at the appropriate duration. Temporal reproduction: participants reproduce a duration after it is presented. Temporal estimation: verbal estimation of the duration. Finger-tapping reproduction task: participants reproduce the clicks/beats by tapping their finger at same pace. Temporal bisection: participants are trained on two anchor durations and presented with intermediate durations. SZ, patients with schizophrenia; SZA, patients with schizoaffective disorder; SPD, schizotypal personality disorder; HC, healthy controls; m, mean; SD, standard deviation; M, male; F, female; DSM, Diagnostic and statistical manual of mental disorders; ICD, International classification of diseases; yr, year; ↓, decrease/slower/under; ↑, increase/faster/over; ns, non-significant differences; N/A, not available/not provided; PFC, prefrontal cortex; DLPFC, dorsal-lateral prefrontal cortex; SMA, supplementary motor area; BG, basal ganglia*.

**Figure 1:**
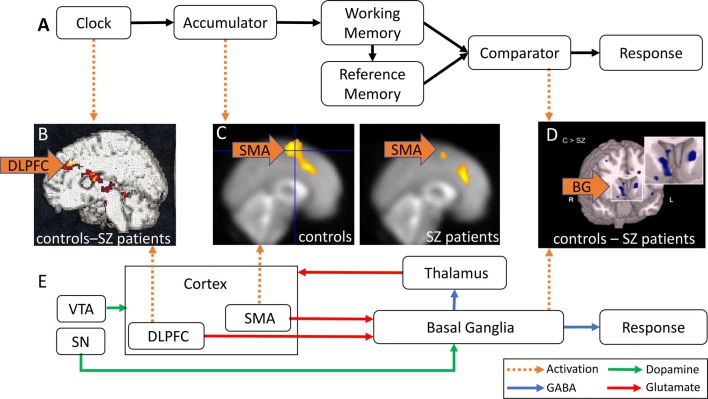
Abnormal activation in brain regions implicated in time perception (TP) in schizophrenia (SZ) patients. **(A)** Information-Processing model of TP (modified from Gibbon et al., 1984). Pulses emitted by an internal clock are stored in working memory (WM) and subsequently encoded in reference memory. The comparator compares the current duration in WM and the stored target duration in reference memory, and triggers responses at the appropriate time. **(B)** DLPFC hypoactivation in SZ patients relative to controls (reproduced with permission from Volz et al., 2001). **(C)** SMA hypoactivation in SZ patients (right panel) relative to healthy controls (HCs; left panel; reproduced with permission from Ortuño et al., 2005). **(D)** Caudate nucleus hypoactivation in SZ patients relative to controls (reproduced with permission from Yoon et al., 2013). **(E)** Striatal Beat Frequency (SBF) model of TP (modified from Buhusi and Meck, 2005; Harrington and Rao, 2015). Cortical oscillations generate oscillatory beat patterns detected and encoded by BG medium spiny neurons. The onset of a timed duration initiates a phasic release in dopamine (DA) from the SN and VTA that synchronizes cortical oscillations in order to encode the to-be-timed duration in the BG. DLPFC, dorsal-lateral prefrontal cortex; SMA, supplementary motor area; VTA, ventral tegmental area; SN, substantia nigra; BG, basal ganglia; green arrows, DA; blue arrows, GABA; red arrows, glutamate; orange arrows, abnormal brain activation.

Figure 1A shows a schematic of a cognitive model of TP, the Information-Processing model (Gibbon et al., 1984). Regular pulses emitted by an internal clock are collected by an accumulator, stored in *working memory* (WM), and encoded for later use in the reference memory. At test, the current duration stored in WM and the duration encoded in reference memory are compared, and a response is made at the appropriate time. In this model, duration overestimation in SZ is explained by a faster clock resulting in greater accumulation of pulses at the time of the response, whereas variability in time estimation in SZ may be due to variability at clock, memory, and decision making levels (Gibbon et al., 1984).

An alternative, neurobiological model of TP, the *Striatal Beat Frequency* (SBF) model (Matell and Meck, 2004; Buhusi and Meck, 2005; Buhusi and Oprisan, 2013), is shown in Figure 1E. The model proposes that the cortico-striatal-thalamo-cortical system mediates cognitive processes involved in TP such as attention and WM (Buhusi and Meck, 2005). Cortical oscillations produce oscillatory beat patterns detected and encoded by striatal medium spiny neurons. The onset of a temporal duration initiates a phasic release of *dopamine* (DA), whereby DA neurons in the *ventral tegmental area* (VTA)/*substantia nigra* (SN) synchronize both cortical oscillations and the membrane levels of striatal medium spiny neurons (Matell and Meck, 2004; Buhusi et al., 2016). Indeed, DA is thought to be a neurotransmitter system crucial for both TP and attention to time (Buhusi and Meck, 2002; Buhusi, 2003). The SBF model is supported by data implicating *dorsal-lateral prefrontal cortex* (DLPFC), the *supplementary motor area* (SMA), the posterior parietal cortex, *basal ganglia* (BG), and the thalamus (Buhusi and Meck, 2005; Ivry and Schlerf, 2008) in TP. In this model, duration overestimation in SZ is explained by an increase in DA activation (equivalent to a faster clock, see Oprisan and Buhusi, 2011), whereas variability in time estimation in SZ may be due to abnormalities in neurotransmission/activation along the cortico-striato-thalamo-cortical loop, as discussed in this review article.

As SZ patients exhibit both impairments in cognitive functioning and TP, it is unclear whether cognitive deficits in attention and WM are responsible for timing deficits in SZ, or disruption of TP is responsible for greater cognitive dysfunction. The current review will address this issue by examining the neural network involved in TP and highlighting abnormalities in these regions in SZ patients and proposes that both TP and cognitive deficits in SZ patients are a result of a malfunction of neurotransmission in the cortico-striatal-thalamo-cortical network.

## Dorsal-Lateral Prefrontal Cortex (DLPFC) and Working Memory for Time

The DLPFC is responsible for attending to presented stimuli and maintaining a representation of a given duration in WM in timing tasks (Curtis and D’Esposito, 2003; Coull et al., 2004). Neuroimaging and *repetitive transcranial magnetic stimulation* (rTMS) studies support a role for the DLPFC in TP. Healthy participants underestimate the to-be-timed duration when rTMS is delivered to the right DLPFC (Koch et al., 2003; Jones et al., 2004), while rTMS delivered to the DLPFC has been shown to improve timing accuracy in Parkinson’s disease (PD) patients (Koch et al., 2004). The effects of rTMS on timing in PD patients may be due to decreases in DLPFC activation due to lower DA levels in comparison to healthy participants. *Functional magnetic resonance imaging* (fMRI) studies indicate greater activation in the DLPFC in healthy participants during timing tasks (Rao et al., 2001; Hinton and Meck, 2004; Üstün et al., 2017). Additionally, the PFC is implicated in TP in animals, both in lesion studies (Buhusi et al., 2018), pharmacological studies (Matthews et al., 2012), and in animal models of SZ (Buhusi et al., 2013).

Very few studies have investigated the neural correlates of TP in SZ patients. Volz et al. (2001) reported DLPFC hypoactivation in SZ patients relative to controls during temporal discrimination (Table 1). Figure 1B shows a contrast in activation between the control and SZ groups after subtraction of resting activation (bright yellow = greater contrast, dark red = smaller contrast). Figure 1B shows that SZ patients exhibited less DLPFC activation than controls, primarily due to DA imbalances in the BG, and secondarily due to abnormalities in the cortico-striatal-thalamo-cortical circuitry which may be responsible for attention and WM deficits (Volz et al., 2001). These findings are consistent with previous research reporting DLPFC hypoactivation in SZ patients in tasks involving sustained attention, such as the *Continuous Performance Test* (CPT), and tasks requiring WM, such as the *Wisconsin Card Sorting Task* (WCST, Weinberger et al., 1986; Barch et al., 2001). Decreased DLPFC activation during these tasks typically correlate with worse performance in patients.

One explanation for hypofrontality in SZ is a dysfunction in *gamma-aminobutyric acid* (GABA), an inhibitory neurotransmitter. Studies in primates indicate an increase in the firing rate of GABAergic DLPFC neurons during a delay in WM tasks (Lewis et al., 2005). Similarly, in the CPT task, decreases in DLPFC activation were observed in SZ patients during a delay presented between a cue stimulus and target stimulus (Barch et al., 2001). These findings indicate that recruitment of the DLPFC, mediated by GABA neurons, is necessary for WM and is disrupted in SZ. Lower GABA levels in the DLPFC in patients may explain hypoactivation in SZ: GABA increases synchronization of pyramidal cell neurons, facilitating task performance and resulting in increased DLPFC activation during WM tasks in healthy participants (Lewis et al., 2005).

## Supplementary Motor Area (SMA) and Temporal Attention

The SMA is involved in the generation of voluntary movements and storage of learned motor actions (Eccles, 1982). Although the SMA is primarily responsible for generating movement, many timing tasks have shown SMA activation when no motor output is required (e.g., Schubotz et al., 2000). Current research suggests that the role of the SMA in TP is to select populations of neurons to mediate temporal attention. Single-cell recording studies in primates have indicated neuronal coding of interval timing in the pre-SMA (Mita et al., 2009) and many studies have reported increased SMA activation during interval timing (Coull et al., 2004; Macar et al., 2004). In the Information-Processing model (Figure 1A), the SMA may act as an accumulator, recording the number of pulses in a given period (Coull et al., 2004). The SMA is also active during tasks involving the processing of rhythms and beat perception (Grahn and Brett, 2007), supporting the SBF model, which proposes that SMA, together with other cortical areas, generates oscillatory input which is detected and encoded in BG medium spiny neurons (Figure 1E).

SZ patients exhibit abnormal SMA activation during timing tasks. Ortuño et al. (2005) used *positron emission tomography* (PET) to examine cerebral blood flow in *healthy controls* (HCs) and SZ patients during a temporal reproduction task (Table 1). Although Ortuño et al. (2005) reported no statistically significant differences in temporal reproduction relative to controls, less SMA activation was reported for SZ patients relative to controls during the timing condition. Figure 1C shows SMA activation (yellow) in the control group (left panel) and SZ group (right panel; Ortuño et al., 2005), indicating that SZ patients exhibit less SMA activation relative to controls, and suggesting dysfunction of the right SMA and prefrontal areas in SZ patients, which may reflect a failure in early time processing related to attentional deficits.

A decrease in SMA activation has been previously reported in SZ patients in both fMRI (Schröder et al., 1995) and *electroencephalography* (EEG) studies (Dreher et al., 1999). For example, SZ patients exhibit an attenuated readiness potential (Dreher et al., 1999). The readiness potential is observed during the planning of motor actions, and source-localization techniques suggest that it is generated in the SMA (Praamstra et al., 1996). SMA recruitment is necessary for facilitating temporal attention, and SZ patients do not actively recruit the SMA during timing tasks, indicated by SMA hypoactivation. As the SMA is responsible for sending information to the BG for motor preparation (DeLong and Wichmann, 2007), it is possible that the SMA encodes a distorted duration before sending it to the BG to be stored in reference memory.

## Basal Ganglia (BG) and Reference Memory for Time

The role of the BG in TP is well-established (Buhusi and Meck, 2005; Buhusi and Cordes, 2011). PD patients show both interval timing and motor timing deficits (e.g., reproducing rhythms by finger-tapping), indicating that BG is necessary for TP (Harrington et al., 1998). Similarly, Huntington’s disease patients, in which BG degeneration occurs, perform worse than HCs on timing tasks (Cope et al., 2014). Increased BG activation during interval timing tasks has also been observed in fMRI scans of healthy participants (Ferrandez et al., 2003; Nenadic et al., 2003). Further, Rao et al. (2001) used event-related fMRI in a temporal discrimination task designed to activate regions in the fronto-striatal network during the encoding of a standard duration and a comparison of a new duration to the standard duration. An early BOLD signal (2.5–5 s after trial onset) associated with encoding a representation of the standard duration was observed in the BG, whereas a late BOLD signal (7.5–10 s after trial onset) associated with the retrieval and comparison of the two durations was observed in the right DLPFC (Rao et al., 2001).

As DA mediates BG functioning (Rammsayer and Classen, 1997), studies have examined the effects of altering DA levels on TP. In the framework of the Information-Processing model (Figure 1A, Gibbon et al., 1984), pacemaker pulses have been described as the firing of DA neurons (Gibbon et al., 1997). Indeed, administration of DA agonists results in a faster clock speed, whereas administration of DA antagonists results in a slower clock speed (Meck, 1996; Buhusi and Meck, 2002).

In the SBF model of TP, DA is assumed to mediate the encoding of temporal durations in the BG, suggesting that a dysfunction in the neuromodulation of DA may be responsible for both timing and other cognitive (e.g., attention, WM) deficits in SZ patients. Indeed, Buhusi (2003) showed that DA has a role both in timing and attention to time. Meyer-Lindenberg et al. (2002) found that PFC hypoactivation in SZ is associated with increased DA levels in the striatum. Relative cerebral blood flow was measured in the DLPFC during the WCST using PET and tracer-6 FDOPA to measure presynaptic DA activity in the striatum (Meyer-Lindenberg et al., 2002). Patients exhibited activity in the DLPFC that was negatively correlated with presynaptic DA activity; the less activation observed in the DLPFC, the greater the striatal DA uptake. This finding is compatible with the finding that increases in striatal DA are associated with a faster internal clock speed, which is often exhibited in SZ patients. This research suggests that timing deficits in SZ may be due to an interaction of increased DA activity in the BG and dysfunction of the DLPFC.

In addition to the DLPFC hypoactivation discussed above, Volz et al. (2001) reported hypoactivation of the caudate nucleus in SZ patients when compared to HCs, and concluded that the cortico-striatal-thalamo-cortical network is disrupted in SZ. If DA hyperactivity of the BG is associated with hypoactivation of the DLPFC in SZ, as observed in Meyer-Lindenberg et al.’s (2002) study, one should expect increases in activation in the BG to be correlated with decreases in DLPFC activation, which is contradictory to Volz et al.’s (2001) findings of hypoactivation in the caudate nucleus. Additional studies report hypoactivation of the caudate nucleus in SZ during WM tasks (e.g., Koch et al., 2008; Yoon et al., 2013). During the response phase of a WM task (Table 1), Yoon et al. (2013) found hypoactivation of the caudate nucleus and reduced functional connectivity between the PFC and the striatum in SZ. Figure 1D shows an fMRI contrast of activation between the control and SZ groups, showing brain regions where activation was greater in controls compared to SZ group (light blue = greater contrast). A large contrast is shown in the caudate nucleus, where patients exhibited hypofunction relative to controls. Functional connectivity between the PFC and striatum was also reduced.

The BG receive input primarily from the PFC and send output to the frontal regions *via* the thalamus (Alexander et al., 1986; Alexander and Crutcher, 1990; Figure 1E). It is therefore unclear whether abnormalities in the cortico-striatal-thalamo-cortical circuits are primarily due to DLPFC dysfunction, or rather dysfunction of the BG. Many studies report that symptoms caused by PFC dysfunction in SZ (e.g., cognitive deficits) occur before positive symptoms (e.g., hallucinations and delusions; Lesh et al., 2011), resulting from hyperactivity in the mesolimbic DA pathway. DA activity is typically increased in the striatum in SZ and decreased in the frontal regions, which suggests less DA is released to the DLPFC during tasks involving WM and attention, reflecting an inability to actively recruit these regions during task performance.

## Implications for Schizophrenia

Frith (1987) proposed that positive symptoms in SZ are a result of abnormalities in the intentions of actions. Patients are unaware of the sources of their actions which results in misattributions of the causes of consequences (e.g., delusions and false beliefs), and a disturbed sense of agency and self (Martin et al., 2017). Patients show a stronger binding of actions and consequences (Haggard et al., 2003), and a stronger binding of separate events in the absence of actions (Franck et al., 2005), judging events as synchronous over larger temporal disparities (Noel et al., 2018). These results suggest that temporal integration of events may lead to misrepresentations of events that are lost (e.g., inability to identify the beginning or end of an action sequence), therefore leading to misattributions. However, there are limitations to these findings, as explicit timing tasks were not employed in all studies.

Voss et al. (2017) identified the angular gyrus and DLPFC as being implicated in matching outcomes to actions and observed decreased connectivity between these regions in SZ. As hypoactivation of the inferior parietal gyri has been observed in SZ patients in TP (Alustiza et al., 2016), future studies should employ binding paradigms (e.g., Haggard et al., 2003) to examine neural activation during TP in SZ. Interestingly, Lošák et al. (2016) found decreased activation of the cerebellar vermis in SZ patients correlated with a faster clock during a time prediction task, suggesting that this region is critical for time prediction during salient events. As many studies report a correlation between a faster clock and positive symptoms in SZ patients, the nature of the relationship between TP and positive symptoms in SZ should be further investigated.

Here, we propose that both timing deficits and cognitive deficits in SZ are a result of neurotransmitter dysfunction in the cortico-striatal-thalamo-cortical loop. As information is transmitted in a loop, dysfunction in one region may cause impairments in another along the loop, rather than disruption occurring in a unidirectional manner. For example, correlations between TP performance and cognitive functioning measures have been observed in SZ (Lee et al., 2009; Roy et al., 2012). Roy et al. (2012) reported greater variability that was negatively correlated with WM performance during a reproduction task; patients with better WM exhibited less variability in the time reproduction task. Neuroimaging may be used to further examine dysfunction in the cortico-striatal-thalamo-cortical circuit and correlations between cognitive functioning and timing. As timing deficits have also been observed in individuals at high risk for SZ (Penney et al., 2005), neuroimaging studies may be used to assess dysfunction in this circuit correlated with TP in first-degree relatives of SZ patients, to assess timing deficits as a potential biomarker for SZ.

## Conclusions

Current research examining the neural correlates of TP in SZ patients is limited. Previous research suggests a disrupted cortico-striatal-thalamo-cortical network which may be responsible for timing deficits observed in SZ. The DLPFC, SMA, and BG play distinct roles in TP, and abnormal activation in these regions is reported in timing tasks as well as additional tasks involving attention and WM in SZ. Timing deficits in SZ may be primarily due to increased DA levels in the BG and less DA and GABA in the DLPFC, which are necessary to mediate WM and attention during TP. Rather than timing deficits in SZ occurring as a result of cognitive dysfunction or vice versa, a malfunction in the neurotransmission of DA and GABA in the cortico-striatal-thalamo-cortical network may be responsible for disruption in the internal clock and cognitive functioning in SZ. As there are very few studies which examine neural activation during TP tasks in SZ, further research is needed to corroborate current findings. Future research should consider potential differences in TP performance due to duration of the illness and the extent to which abnormalities in neural activation during timing tasks are correlated with positive symptom severity, as disruptions in the internal clock rate may be linked to positive symptoms exhibited in SZ. Lastly, future research including neuroleptic-naive patients is necessary to rule out the potential confounds of antipsychotic medication effects on TP (Table 1).

## Author Contributions

Authors contributed equally to all aspects of developing and writing this manuscript.

## Conflict of Interest Statement

The authors declare that the research was conducted in the absence of any commercial or financial relationships that could be construed as a potential conflict of interest.
